# Speech recognition with a hearing-aid processing scheme combining beamforming with mask-informed speech enhancement

**DOI:** 10.1177/23312165211068629

**Published:** 2022-01-05

**Authors:** Tim Green, Gaston Hilkhuysen, Mark Huckvale, Stuart Rosen, Mike Brookes, Alastair Moore, Patrick Naylor, Leo Lightburn, Wei Xue

**Affiliations:** 1 Department of Speech, Hearing and Phonetic Sciences, 4919 UCL, London, UK; 2 Department of Electrical and Electronic Engineering, 4615 Imperial College, London, UK

**Keywords:** Hearing loss, binaural hearing, cocktail party listening, spatial hearing

## Abstract

A signal processing approach combining beamforming with mask-informed speech enhancement was assessed by measuring sentence recognition in listeners with mild-to-moderate hearing impairment in adverse listening conditions that simulated the output of behind-the-ear hearing aids in a noisy classroom. Two types of beamforming were compared: binaural, with the two microphones of each aid treated as a single array, and bilateral, where independent left and right beamformers were derived. Binaural beamforming produces a narrower beam, maximising improvement in signal-to-noise ratio (SNR), but eliminates the spatial diversity that is preserved in bilateral beamforming. Each beamformer type was optimised for the true target position and implemented with and without additional speech enhancement in which spectral features extracted from the beamformer output were passed to a deep neural network trained to identify time-frequency regions dominated by target speech. Additional conditions comprising binaural beamforming combined with speech enhancement implemented using Wiener filtering or modulation-domain Kalman filtering were tested in normally-hearing (NH) listeners. Both beamformer types gave substantial improvements relative to no processing, with significantly greater benefit for binaural beamforming. Performance with additional mask-informed enhancement was poorer than with beamforming alone, for both beamformer types and both listener groups. In NH listeners the addition of mask-informed enhancement produced significantly poorer performance than both other forms of enhancement, neither of which differed from the beamformer alone. In summary, the additional improvement in SNR provided by binaural beamforming appeared to outweigh loss of spatial information, while speech understanding was not further improved by the mask-informed enhancement method implemented here.

## Introduction

Understanding speech in the presence of competing sounds is particularly challenging for hearing-impaired (HI) listeners (Marrone, Mason and Kidd, 2008) and difficulties with speech in noise are a major factor limiting satisfaction with and usage of hearing aids (HAs) (Kochkin, 2010). Signal processing approaches that have been employed to try to improve the perception of speech in noise via HAs include both the use of spatial filtering (beamforming) to emphasise sounds from the direction of the target source and methods that attempt to separate target and masking sounds based on their spectrotemporal characteristics, such as binary masking (Kim and Loizou, 2010). While such approaches can lead to substantial improvements in speech perception in ideal laboratory conditions, the benefit afforded to HA users in real-world situations may be more limited (e.g., Wu *et al.*, 2019). Factors that may limit real world benefit include the absence of a priori knowledge of the target and masker characteristics, the limited time available for processing, the need to preserve a natural sound quality and the fact that real world listening is typically dynamic with moving sound sources and listeners moving their heads.

However, advances in technology have provided possibilities for improved signal processing approaches. These include the potential for bilateral HAs to exchange information and the use of deep neural networks (DNNs) to aid the separation of speech from noise (e.g., Kolbaek, Yu, Tan & Jensen, 2017; Luo & Mesgarani, 2019; Xu, Du, Dai & Lee, 2015). A major focus of current research concerns how to integrate techniques such as spatial filtering and speech enhancement (e.g., Andersen et al, 2021; Koutrouvelis, Hendriks, Heusdens & Jensen, 2017). The ultimate goal is to raise the signal-to-noise-ratio (SNR) of target speech as much as possible while maintaining sufficient flexibility to accommodate changes in the target source and preserving a natural seeming sound environment, including spatial information. In this study we evaluated a number of processing schemes which perform co-ordinated signal processing on signals obtained from a pair of bilateral HAs, with each aid having two microphones. Various combinations of beamforming and additional enhancement in the time-frequency (TF) domain were tested.

Beamforming makes use of multiple microphone signals to attenuate sound energy selectively from particular directions, enabling an increase in SNR when target speech is sufficiently spatially separated from noise sources. Earlier laboratory studies, in which there was no exchange of information between HAs, have demonstrated that beamforming gives substantial benefits over the use of omnidirectional microphones (Bentler, Palmer, & Mueller, 2006; Luts, Maj, Soede, & Wouters, 2004; Saunders & Kates, 1997). However, benefits are reduced in more realistic conditions, including when reverberation is present (Ricketts & Hornsby, 2003). One approach to combining information from the two HAs is to treat all the available microphones as a single array to create a binaural beamformer. This enables a narrower beam to be formed and so maximises the potential increase in SNR, provided that the beam can be steered with sufficient accuracy. In this approach, however, all the residual noise is co-located with the target speech and it is not possible for the listener to utilise spatial cues for the localisation and segregation of different sound sources. In bilateral beamforming, in contrast, beamformers are implemented separately in the left and right channels, each derived from the pair of microphones on a single HA. In this case the spatial diversity of the residual noise is preserved but there is less noise attenuation because the beam is broader. A variety of different approaches have been implemented that attempt to balance the increased SNR provided by a narrower beam with the preservation of spatial cues in the noise background (Best, Roverud, Mason, & Kidd, 2017; Doclo, Kellermann, Makino, & Nordholm, 2015; Gössling, Marquadt & Doclo, 2020; Kidd, Mason, Best, & Swaminathan, 2015; Neher, Wagener, & Latzel, 2017; Wang, Best, & Shinn-Cunningham, 2020).

Here, however, we implemented standard binaural and bilateral minimum variance distortionless response (MVDR) beamformers optimised for the true target position and focused on examining the effect of applying additional speech enhancement in the TF domain. Three different methods of determining the gains for each TF region were implemented. The primary approach was adapted from the principle of binary masking (Kjems, Boldt, Pedersen, Lunner, & Wang, 2009) and involved output from the beamformer being fed to a DNN trained to identify TF regions containing significant target speech energy. Speech recognition of HI listeners was assessed for bilateral and binaural beamformers both with and without additional speech enhancement. Performance was tested in a simulated noisy school classroom to provide a reasonably realistic representation of the adverse listening conditions that are particularly challenging for HI listeners. Additional testing carried out in normally-hearing (NH) listeners implemented two alternative forms of speech enhancement: Wiener filtering (Gerkmann & Hendriks, 2012) and modulation-domain Kalman filtering (Wang & Brookes, 2018). Both HI and NH listeners were also tested in a small set of conditions in a simulated anechoic environment. The main goals of the study were to compare the benefit provided to HI listeners by binaural and bilateral beamformers and to assess whether further benefit could be achieved by implementing mask-informed speech enhancement in combination with beamforming.

## Methods

### General

Testing took place in a sound-proofed booth and was controlled by custom MATLAB software running on a Dell Latitude E6440 computer. Stimuli were presented via a RME Fireface 400 audio interface and Sennheiser HDA-200 headphones.

### Simulated Test Environments

An anechoic chamber was simulated using binaural impulse responses from a database provided by Kayser et al. (2009). These responses were recorded from the microphones of behind-the-ear (BTE) HAs mounted on a head-and-torso simulator which was 0.8 m away from the loudspeaker (measured to the centre of the simulator's head). In the signal processing conditions that implemented beamforming, responses from the front and back microphone of each HA were used. In the unprocessed condition, only responses from the front microphones were used. The responses were used to simulate situations in which target speech was always presented from 0˚ while a speech-shaped noise masker was presented from either 0˚ or 60˚ to the right. Speech recognition performance typically improves when target and masker are spatially separated and the difference between performance with the two noise locations provides a measure of spatial release from masking (SRM).

To allow simulation of a classroom environment we recorded noise and binaural room impulse response (BRIR) measurements in a primary school. These recordings were made using dummy BTE HAs placed on a KEMAR manikin (45BB-1) equipped with soft large pinnae (GRAS KB1065 & KB1066). Each dummy HA contained two microphones in their typical casing, but none of the other components found in functioning HAs. Signals were captured from the four microphones available in the two HAs placed in a standard position on the manikin's ears.

**Figure 1.**
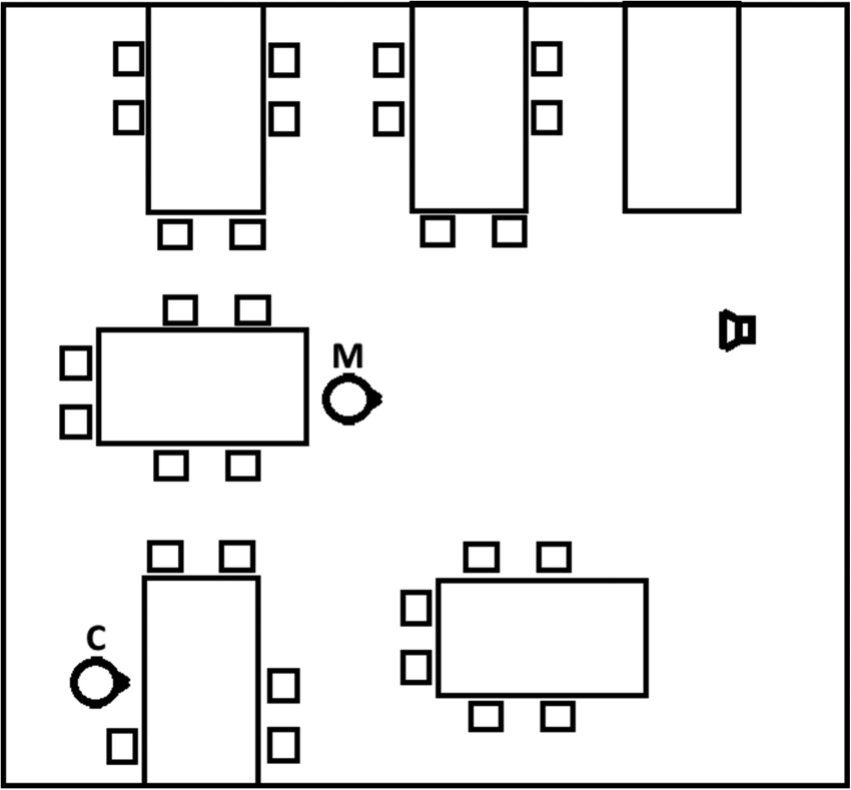
A schematic representation of the school classroom, showing the location of the loudspeaker and the corner (C) and middle (M) locations from which KEMAR recordings were made. The small squares indicate the approximate positions of the children.

The dimensions of the classroom were 7.2×6.7×3.3 m and it had a T_60_ of 0.42 s. Three of the walls were standard brick, while one of the longer walls contained several large windows. A Fostex 6301B loudspeaker, which has similar directivity to that of a human voice (Luizard, Brauer, Weinzierl, & Bernadoni, 2018), was placed at the front of the room in a position typically occupied by the teacher. Recordings were made with the KEMAR in two different room locations: near the middle and near the back corner on the side opposite to the windows. Figure 1 shows a schematic representation of the room layout. The HA microphones and the centre of the loudspeaker were 1.1 and 1.7 m from the floor, respectively.

The class contained approximately 30 children aged 7 or 8, as well as their teacher. Ambient noise was recorded for 300 seconds while the children played a mathematical game that involved rolling dice. They collaborated within small groups, so that there were multiple concurrent voices along with other incidental noises, most noticeably the rolling dice. BRIRs were recorded with the children in place but sitting quietly, while the loudspeaker emitted a 20-s chirp exponentially increasing in frequency from 62.5 to 8000 Hz. The chirp included a compensation for the loudspeaker's frequency response. BRIRs and noise recordings were used to simulate conditions in which target speech at the loudspeaker position was presented to a listener at either the corner (C) or middle (M) location.

### Signal Processing

**Figure 2.**
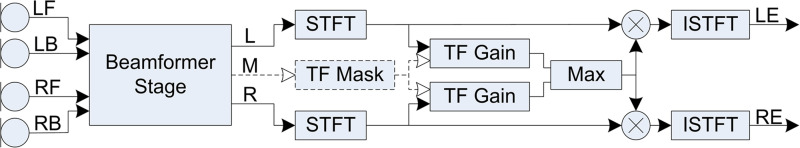
Block diagram of data processing.

A block diagram of the data processing is shown in Figure 2. LF and LB denote the front and back microphone signals from the left HA while RF and RB denote the corresponding signals from the right HA. The beamformer stage combined these microphone signals to generate directionally selective signals for left (L) and right (R) channels, along with an additional directionally selective signal (M) used in estimating a TF mask. Subsequent stages then applied enhancement processing in the TF domain to generate the enhanced left and right output signals, LE and RE.

*Beamforming.* The beamformer stage applied finite impulse response filters to the four microphone signals and added their outputs to obtain the directionally selective signals, L, R and M. Three configurations were implemented. In the first configuration, no beamformer was used and the L and R outputs of this stage were taken directly from the front microphones, LF and RF. In the second configuration, a binaural beamformer (BN) was designed for the left (L) and right (R) channels using all four input microphone signals in each case. The design implemented an MVDR beamformer (Bitzer & Simmer, 2001) and used calibration recordings of the target speaker and of the background noise to obtain the required design parameters. Using a calibration recording of the target speaker in this way ensured that the beamformer was optimised for the true target position even though the KEMAR was positioned to look straight ahead in our experiments. This beamformer minimises the background noise power subject to the constraint that the target speaker signal at the reference microphone passes unaltered to the output signal. The reference microphone was chosen to be LF for the L output and RF for the R output. In the third configuration, a bilateral beamformer (BL) was designed for each of the left (L) and right (R) channels. This was also an MVDR beamformer, but instead of using all four microphone signals, each beamformer used only two signals: LF and LB for the L output or RF and RB for the R output. The BL configuration provides less directional selectivity than the BN configuration but approximately retains the spatial cues of the noise (Kuklasinski & Jensen, 2017). Comparing these two configurations allows the relative benefit of SRM to be compared with enhanced directional selectivity.

One of the enhancement regimes described below incorporated a TF mask that identified the TF regions containing significant energy from the target speaker. This mask was estimated from a separate beamformer output (M) designed as a binaural MVDR beamformer using LF as the reference microphone. Thus the M and L outputs were identical in the BN configuration but were different in the BL configuration.

*Mask-informed Enhancement.* Speech enhancement was applied to the beamformer output signals by applying a different multiplicative gain in each TF region. Typically, the gain is close to zero in regions that are heavily corrupted by noise and close to unity in regions that are noise-free. To perform the enhancement, the signals were first converted into the TF domain using the short-time Fourier transform (STFT). After applying an appropriate gain in each TF region, the signals were converted back into time waveforms using the inverse STFT (ISTFT, Crochiere, 1980). The STFT used 75%-overlapping 16 ms frames, so each TF region corresponds to 4 ms × 62.5 Hz. In order to preserve spatial cues, it is necessary to ensure that the left and right channels are multiplied by the same gains. Accordingly, in each TF region the enhancement algorithm calculated separate gains for the left and right channels but then applied the larger of these two gains to both channels. Since a larger gain is associated with lower noise levels, this is approximately equivalent to selecting the gain appropriate to the ear with the better SNR and applying it to both channels.

Three alternative enhancement regimes were implemented, differing only in how the TF gain was calculated from the beamformer output signals. The first enhancement regime (M) used an estimated mask to control the TF gain (Moore, Lightburn, Xue, Naylor, & Brookes, 2018; Brookes, Lightburn, Moore, Naylor, & Xue, 2019). In this algorithm, spectral features extracted from the M output of the beamformer form the inputs to a DNN which has been trained to identify the TF regions that contain significant target speech energy. An overview of the mask estimation procedure is given below; full details of the neural network structure, input features and training regime are given in Moore et al. (2018) and Lightburn (2020). The input features of the neural net were derived from a 90-channel cochleagram calculated using 25.6 ms frames with 50% overlap. The cochleagram outputs from a sliding window of 13 consecutive frames were concatenated to provide the input of the neural net. The neural net output was a continuous-valued mask estimate for 129 uniformly spaced frequency bins for each of five consecutive frames. The five estimates of each mask value arising from successive input window positions were averaged to obtain the final mask value in the range [0,1]. For each speech utterance in the training set, the training target of the neural network was the TF binary mask that, when the input utterance was corrupted with additive white Gaussian noise at -5 dB SNR, maximised the expected value of a speech intelligibility metric. The advantages of using this mask for the training target rather than the more commonly used ideal binary mask are that it automatically adapts to temporal variations in speech energy, avoids the need for an arbitrarily-chosen local criterion and does not depend on a specific realisation of the noise. The speech intelligibility metric used for the mask optimisation was the WSTOI metric from Lightburn et al. (2016), modified to use 129 uniformly spaced frequency bins. The output of the TF mask estimator was used to condition a trained statistical model of the normalised complex-valued speech spectral amplitudes. The model used was a two-component zero-mean complex Gaussian mixture model and the normalisation factor was the active speech level estimate from ITU-T P.56 (1993). Finally, this model was used as the prior speech distribution in an enhancer that minimises the mean square error of log spectral amplitudes (Ephraim & Malah, 1985). The second enhancement regime (W) used a Wiener filter gain given by the estimated ratio of the power of the target speaker in each TF region to that of the noisy signal. The power of the target speaker in each TF region was deduced by using the algorithm from Gerkmann and Hendriks (2012) to estimate the power of the background noise. The third enhancement regime (K) used the algorithm described in Wang and Brookes (2018) which incorporates a Kalman filter in each frequency band to model the temporal evolution of the speech amplitude spectrum from which the required gain in each TF region is determined. Note that the M output of the beamformer in Figure 2 is used only for mask estimation and that the L and R outputs are used by all three enhancement regimes when calculating and applying the TF gains.

### Participants

**Figure 3.**
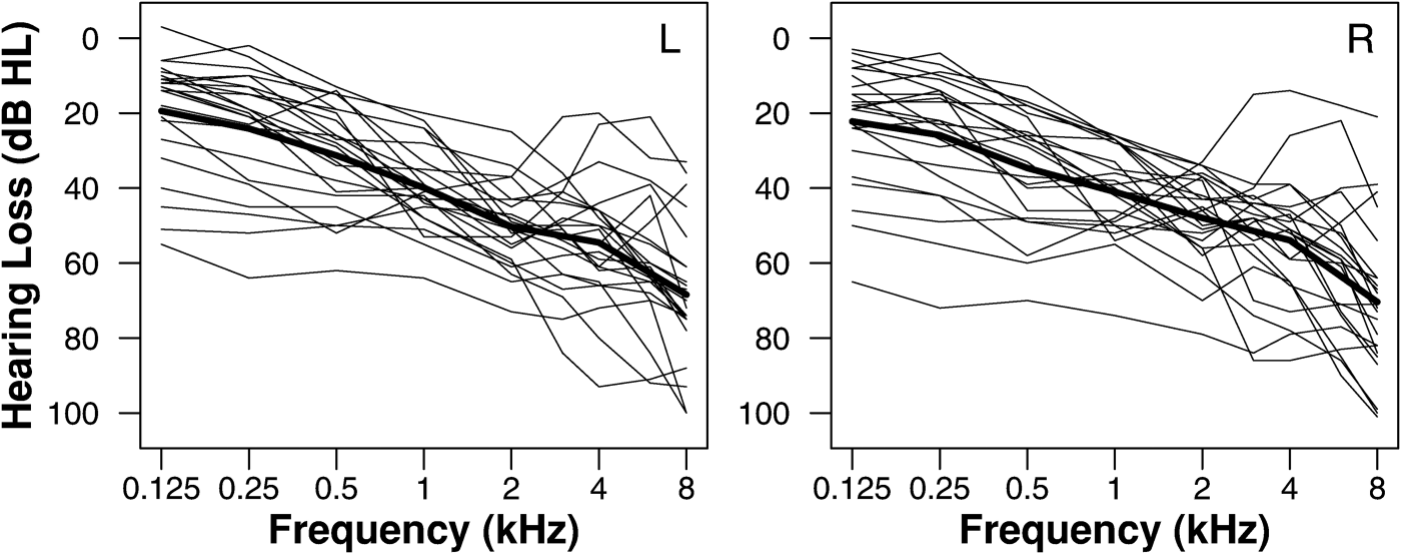
Audiograms of hearing-impaired listeners. Thin lines show individual data and the thick lines show the means. Left and right panels show data from the corresponding ear.

*Hearing-impaired Listeners.* 23 listeners with bilateral sensorineural hearing loss were recruited via UCL Hospital's Audiology department (14 female, 9 male). Ages ranged between 48 and 84 (mean = 72.1). Audiograms are shown in Figure 3. Pure tone thresholds averaged across octave frequencies between 250 and 4000 Hz varied between 25 and 71 dB HL. The difference in mean thresholds across ears was less than 10 dB in all except one case, where it was 10.6 dB. All HI listeners had previously been tested on various psychoacoustic and speech in noise tasks.

*Normal Hearing Listeners.* NH listeners were recruited from the UCL Psychology subject pool, which contains members of the general public as well as staff and students. A total of 28 participated (15 female, 13 male). Ages ranged between 19 and 48 (mean = 25.5). All had pure-tone thresholds of 20 dB HL or better at octave frequencies between 250 and 8,000 Hz. All 28 were tested in anechoic conditions. Different groups of 14 were tested in either the C or M classroom locations.

All participants had British English as their first language and gave informed written consent. Ethical approval was provided by the National Health Service Health Research Authority (Ref: 17/LO/0151).

### Speech Tests

*Materials.* Target speech comprised Institute of Electrical and Electronical Engineers (IEEE) sentences (IEEE, 1969) recorded from a male talker of Standard Southern British English. Each sentence contained five key words on which scoring was based. In anechoic conditions, the spectrum of the speech-shaped noise maskers prior to convolution with the BRIRs matched the average spectrum of the target speech (calculated across the full set of 720 IEEE sentences). In classroom conditions, the noise level varied considerably over time. In order to prevent these fluctuations from introducing undue variability into sentence intelligibility measurements, an automatic gain control (AGC) with a large time constant was used to equalise the level of the classroom recordings over time. The AGC consisted of a sliding rectangular 1-s window, that provided the envelope of the signal from each of the four HA microphones. These envelopes, expressed in log energies, were averaged across the four microphones. We next subtracted this microphone-averaged envelope from its average level over time, resulting in a time varying gain function. The original noise recordings were subsequently time aligned with this gain function such that the AGC had half-a-second look ahead, and the gain was applied to all recorded signals. Both anechoic and classroom environments used randomly drawn noise segments. On each trial the masker began 900 ms before the onset of the target sentence and finished 900 ms after target offset. 100 ms raised-cosines were applied to masker onset and offset.

*Test Conditions.* Seven different processing types were implemented in total, but not all were tested with each combination of listener group and environment. For HI listeners in the classroom environment, five types of processing were tested: unprocessed (*None*), bilateral beamformer only (*BL*), binaural beamformer only (*BN*), bilateral beamformer plus mask-informed enhancement (*BL+M*), and binaural beamformer plus mask-informed enhancement (*BN+M*). Each HI listener was tested with all five types in both the C and M locations. With NH listeners, different groups were tested in the C and M locations but two additional processing types were tested: binaural beamformer plus Wiener filter (*BN+W*), and binaural beamformer plus Kalman filter (*BN+K*). In the anechoic environment, all NH and HI listeners were tested with two types of processing (*None* and *BN+M*), crossed with two noise azimuths (0˚ and 60˚). In total, therefore, each HI listener was tested in 14 conditions and each NH listener was tested in 11 conditions. For HI listeners, frequency-selective compensation for hearing loss (based on the NAL-R formula, Byrne & Dillon, 1986) was applied in all conditions. No attempt was made to mimic the direct low frequency sound that would typically be available to some extent from wearable HAs and which might provide useful binaural cues to some listeners.

*Procedure.* From each listener in each condition, two estimates of the speech reception threshold (SRT) were obtained and averaged. The SRT was defined as the SNR at which 50% of key words were correctly identified and each estimate was derived from responses to a list of 10 sentences. After each sentence the participant said what words they thought they had heard and the experimenter marked each key word.

Presentation levels were controlled by different rules for positive and negative SNRs. At 0 dB SNR both the noise and the target speech were at 65 dB SPL. For negative SNRs the noise level was fixed and the level of the target speech was reduced. For positive SNRs the target speech stayed at 65 dB and the noise level was reduced. The SNR for the first sentence in each run was selected on the basis of pilot data so as to be towards the higher end of the range of likely SRTs for the given condition. The adaptive procedure followed a rule in which both the direction and size of the change in SNR varied according to the number of key words correctly identified. For 0, 1 or 2 correct words, the SNR was increased by 3, 2 or 1 dB respectively. For 3, 4, or 5 correct words, the SNR was decreased by 1, 2 or 3 dB respectively. If no words were correctly identified on the first sentence of a list, that same sentence was repeated with the SNR increased by 3 dB. The mean of the SNRs from the fifth sentence onwards (including the SNR that would have been used for an 11th sentence) was taken as the SRT estimate for each run.

The order in which conditions were completed was blocked by type of environment/location and all conditions were completed once before any were repeated. The order in which types of environment/location (2 for NH listeners, 3 for HI listeners) were completed was counterbalanced across listeners. Within each environment/location block, the order of conditions was also counterbalanced, but the order in which sentence lists were used was the same across listeners. Counterbalancing was based on randomised Latin Squares, with a different randomisation used for the second runs of each condition.

## Results

### Anechoic

**Figure 4.**
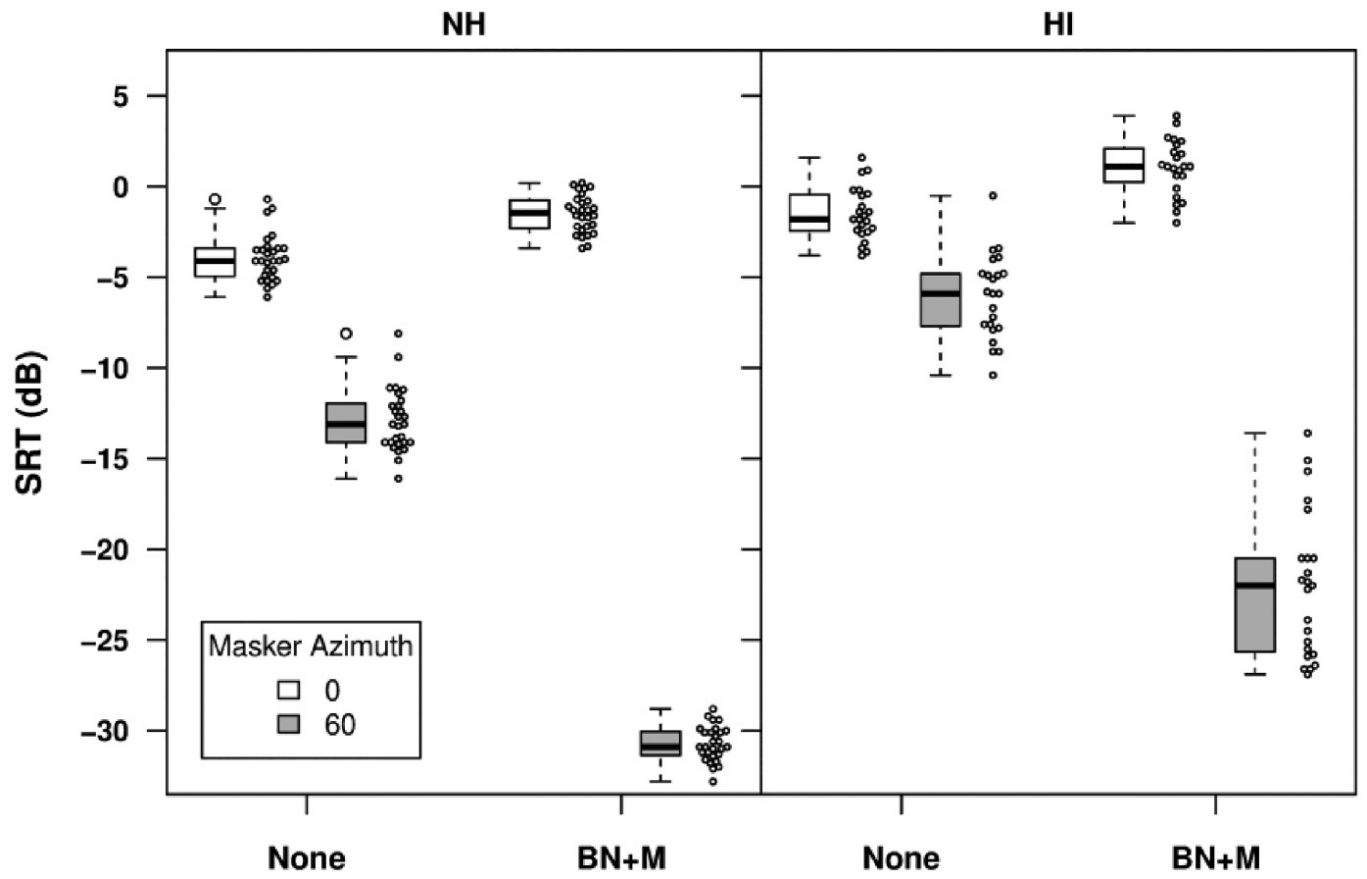
SRTs in anechoic conditions with a single speech-shaped noise masker for normally-hearing (left) and hearing-impaired (right) listeners. The circles to the right of each box show individual data points.

*Normal Hearing listeners.* Figure 4 (left) shows SRTs for all 28 NH listeners in anechoic conditions. For unprocessed stimuli, the mean SRT was -3.9 dB with target speech and noise co-located at 0° azimuth and -12.9 dB with noise at 60°, so that SRM was 9.0 dB. As might be expected in anechoic conditions with a single noise source, applying the combination of binaural beamformer and mask-informed speech enhancement was highly beneficial for spatially separated noise, with the mean SRT improving to −30.7 dB and the mean SRM increasing to 29.2 dB. Note however, that a small part of the increase in SRM arose from the fact that for co-located speech and noise the mean SRT was 2.4 dB poorer with the combined processing applied. Repeated measures ANOVA with factors of noise azimuth and processing type confirmed that both main effects and the interaction were highly significant (Table 1). A paired t-test showed that the decline in performance in the BN+M condition for co-located speech and noise was significant [*t*(27) = 11.99, *p* < 0.001].

**Table 1.** Repeated Measures ANOVA on SRTs of normally-hearing listeners in anechoic conditions. Significant p values at the 5% level are shown in bold.

Factor	df	F	p
Processing Type	(1,27)	1365.27	**<0.001**
Noise Azimuth	(1,27)	12213.31	**<0.001**
Processing Type x Noise Azimuth	(1,27)	5403.70	**<0.001**

*Hearing-impaired listeners.* Figure 4 (right) shows SRTs for HI listeners in the simulated anechoic environment. For unprocessed stimuli with target and noise co-located, the mean SRT was -1.5 dB, somewhat poorer than the -3.9 dB found with NH listeners. The SRM for unprocessed stimuli ranged between 1.3 and 7.7 dB, with a mean of 4.5 dB, half the value observed with NH listeners.

These data are broadly consistent with previous findings in similar conditions. For co-located target and noise the mean deficit relative to NH listeners of 2.4 dB found here for listeners with symmetrical impairment compares to previous deficits of 2.5 dB (Bronkhorst and Plomp, 1989) and 5.4 dB (Duquesnoy, 1983). The 9.0 dB of SRM for NH listeners with noise shifted by 60° in the present study compares with values of 10.1 dB (Bronkhorst & Plomp, 1989) and 9.6 dB (Duquesnoy, 1983) for noise shifted by 90°. For HI listeners, the mean 4.5 dB of SRM found here falls between the 2.5 dB found by Duquesnoy (1983) and values of 7.1 dB (Bronkhorst & Plomp, 1989) and 5.5 dB or 6.5 dB for different groups of HI listeners in Festen & Plomp (1986). Differences across studies in the age of the HI listeners and the extent of their hearing loss, particularly in the higher-frequency range important for headshadow, may contribute to the observed differences in SRM.

With processed stimuli, the mean SRM was 23.1 dB. Similar to NH listeners, the mean SRT for co-located speech and noise was poorer by 2.6 dB with processing applied. Repeated measures ANOVA with factors of noise azimuth and processing type confirmed that both main effects and the interaction were highly significant (Table 2). A paired t-test showed that performance with co-located speech and noise was significantly poorer in the BN+M condition than in the unprocessed condition [*t*(22) = 9.01, *p* < 0.001].

**Table 2.** Repeated Measures ANOVA on SRTs of hearing-impaired listeners in anechoic conditions. Significant p values at the 5% level are shown in bold.

Factor	df	F	p
Processing Type	(1,22)	895.99	**<0.001**
Noise Azimuth	(1,22)	389.20	**<0.001**
Processing Type x Noise Azimuth	(1,22)	773.22	**<0.001**

The effect of hearing impairment on SRM was assessed with a mixed ANOVA with factors of hearing status (normal or impaired) and processing type. This analysis confirmed that SRM was significantly lower for HI users [*F*(1,49) = 131.47, *p*<0.001] and also showed a significant interaction between hearing status and processing type [*F*(1,49) = 6.23, *p* = 0.016], indicating that the increase in SRM for the BN+M condition over the unprocessed condition was significantly smaller for HI listeners.

### Classroom

**Figure 5.**
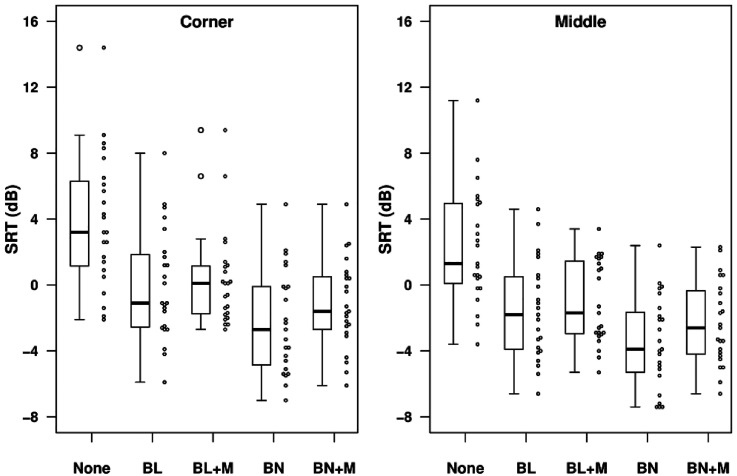
SRTs for each processing condition for hearing-impaired listeners in the classroom environment. The different panels show data for the corner (left) and middle (right) locations. The circles to the right of each box show individual data points.

*Hearing-Impaired listeners.* Figure 5 shows the SRTs for each processing type in the simulated classroom environment for locations C (left panel) and M (right panel). Performance was generally slightly worse overall for location C, but the pattern of performance across conditions was similar for both locations. The mean SRTs for unprocessed stimuli were 3.9 dB for location C and 2.3 dB for location M. Unprocessed SRTs varied widely across individual HI listeners, ranging from −2.1 to 14.4 dB for location C and from -3.6 to 11.2 dB for location M. For both locations, applying a beamformer with no mask-informed enhancement led to mean improvements in SRT relative to the unprocessed condition of around 4 dB for the bilateral case and around 6 dB for the binaural case. Performance with both beamforming and mask-informed enhancement applied was poorer than with beamforming alone, although the differences in mean SRT were small, ranging from 0.3 dB for the bilateral beamformer in location C to 1.2 dB for the binaural beamformer in location M.

**Figure 6.**
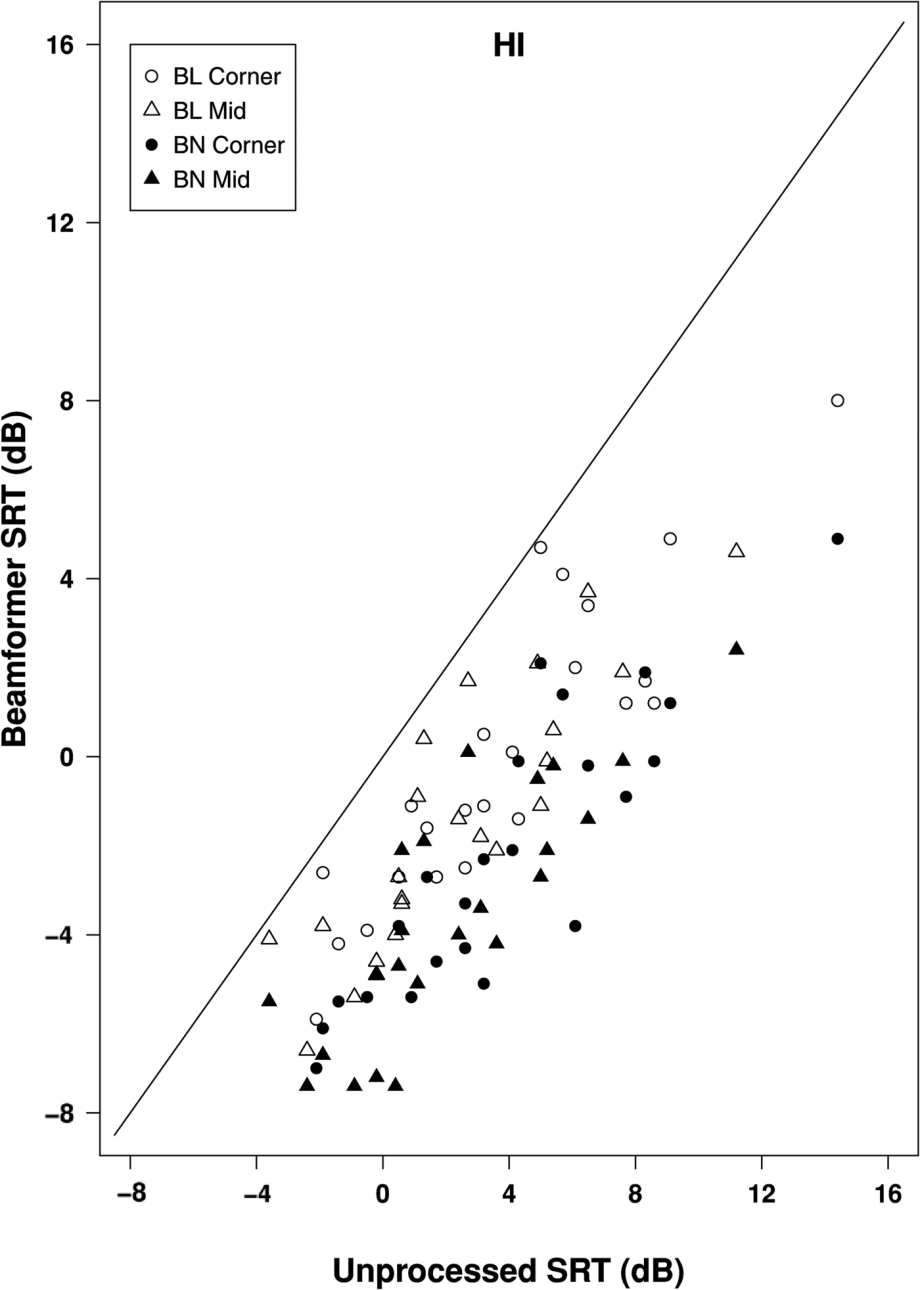
Scatterplot of SRTs in the classroom environment for hearing-impaired listeners showing performance in beamformer alone conditions plotted against that in unprocessed conditions. Points below the line of equality indicate a beneficial effect of beamforming.

Figure 6 shows a scatterplot of SRTs with beamformer alone against SRTs in the unprocessed condition. Open symbols show bilateral beamforming and closed symbols show binaural beamforming. All points lie below the line of equality so that beamforming was always beneficial relative to performance in the unprocessed condition, although the amount of benefit varied considerably across individual cases and was marginal for bilateral beamforming in some instances. The benefit ranged between 0.3 and 7.4 dB for bilateral beamforming and between 1.9 and 9.9 dB for binaural beamforming. In the large majority of individual cases (44 out of 48), for the pair of points at a particular unprocessed SRT the filled symbol lies below the open symbol, indicating a larger benefit for binaural than bilateral beamforming.

**Figure 7.**
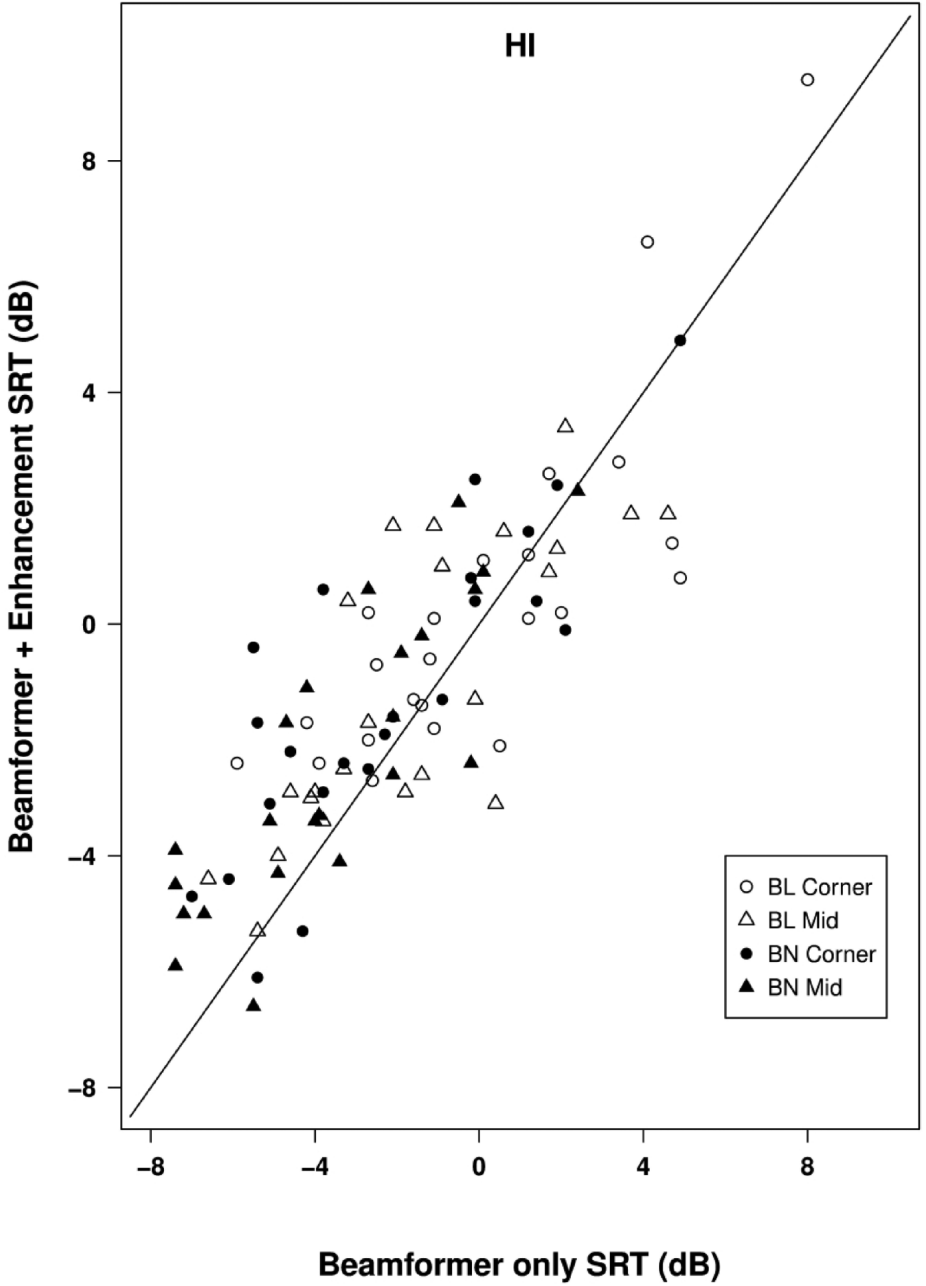
Scatterplot of SRTs in the classroom environment for hearing-impaired listeners showing performance with beamformer plus mask-informed enhancement plotted against that with beamformer alone. Points below the line of equality indicate a beneficial effect of speech enhancement.

Figure 7 illustrates how the effect of adding mask-informed enhancement to beamforming varied across individual cases, showing a scatterplot of SRTs with both beamforming and mask-informed enhancement applied against SRTs with beamformer alone. The majority of the points fall above the line of equality, indicating poorer performance with additional enhancement than with beamformer alone, particularly for binaural beamforming. For binaural beamforming the effect of additional enhancement ranged from a decrease in SRT of 2.2 dB to an increase in SRT of 5.1 dB, while for bilateral beamforming the range was from a decrease of 4.1 dB to an increase of 3.8 dB.

Repeated measures ANOVA treating all five processing conditions as levels of a single factor showed a significant effect of location [*F*(1,22) = 47.89, *p* < 0.001], a significant effect of processing [*F*(4,88) = 114.49, *p* < 0.001] and no significant interaction [*F*(4,88) < 1]. Bonferroni-corrected pairwise comparisons showed that all processing conditions differed significantly from each other with the exception of the bilateral beamformer alone and the bilateral beamformer with mask-informed enhancement.

In order to examine any possible interaction between the type of beamforming and the addition of mask-informed enhancement, the unprocessed condition was omitted and the remaining data were subjected to a three-way repeated measures ANOVA with factors of location, type of beamforming and whether or not enhancement was applied (Table 3). All three main effects were significant and there was also a significant interaction between beamformer type and enhancement. Separate two-way ANOVAs with factors of location and enhancement for each beamformer type showed that the decrease in performance with mask-informed enhancement was significant only for binaural beamforming.

**Table 3.** Repeated Measures ANOVA on SRTs of hearing-impaired listeners in processed conditions in the simulated classroom. Significant p values at the 5% level are shown in bold.

Factor	df	F	p
3-way ANOVA			
Beamformer Type	(1,22)	86.71	**<0.001**
Enhancement	(1,22)	12.02	**0.002**
Location	(1,22)	37.32	**<0.001**
Beamformer Type x Enhancement	(1,22)	6.83	**0.016**
Beamformer Type x Location	(1,22)	0.52	0.477
Enhancement x Location	(1,22)	0.18	0.677
Beamformer Type x Enhancement x Location	(1,22)	0.01	0.945
Binaural Beamformer 2-way ANOVA			
Enhancement	(1,22)	23.62	**<0.001**
Location	(1,22)	22.38	**<0.001**
Enhancement x Location	(1,22)	0.07	0.794
Bilateral Beamformer 2-way ANOVA			
Enhancement	(1,22)	1.76	0.198
Location	(1,22)	21.40	**<0.001**
Enhancement x Location	(1,22)	0.11	0.740

**Figure 8.**
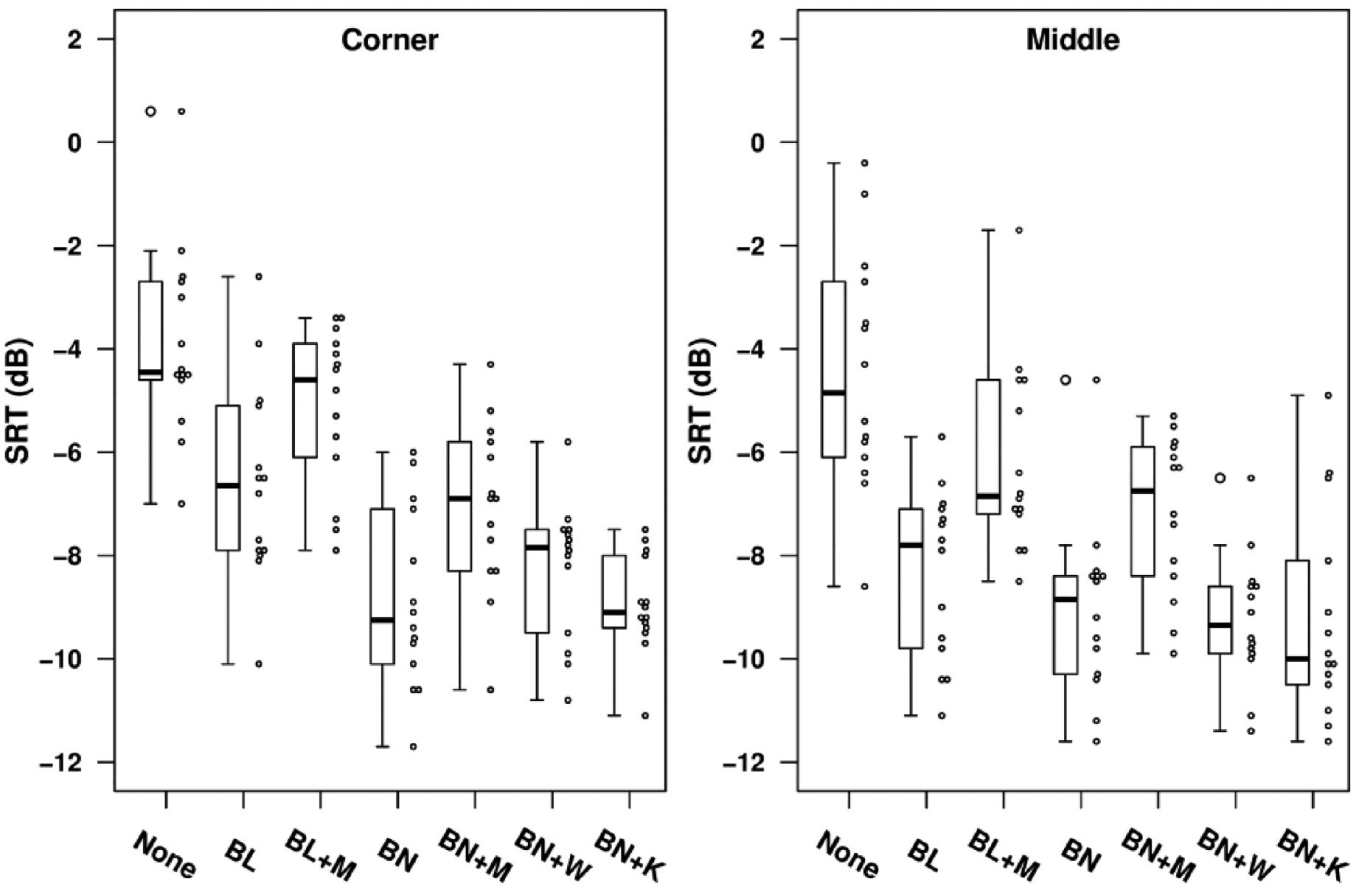
SRTs for each processing condition for normally-hearing listeners in the classroom environment. The different panels show data for the corner (left) and middle (right) locations. The circles to the right of each box show individual data points.

**Figure 9.**
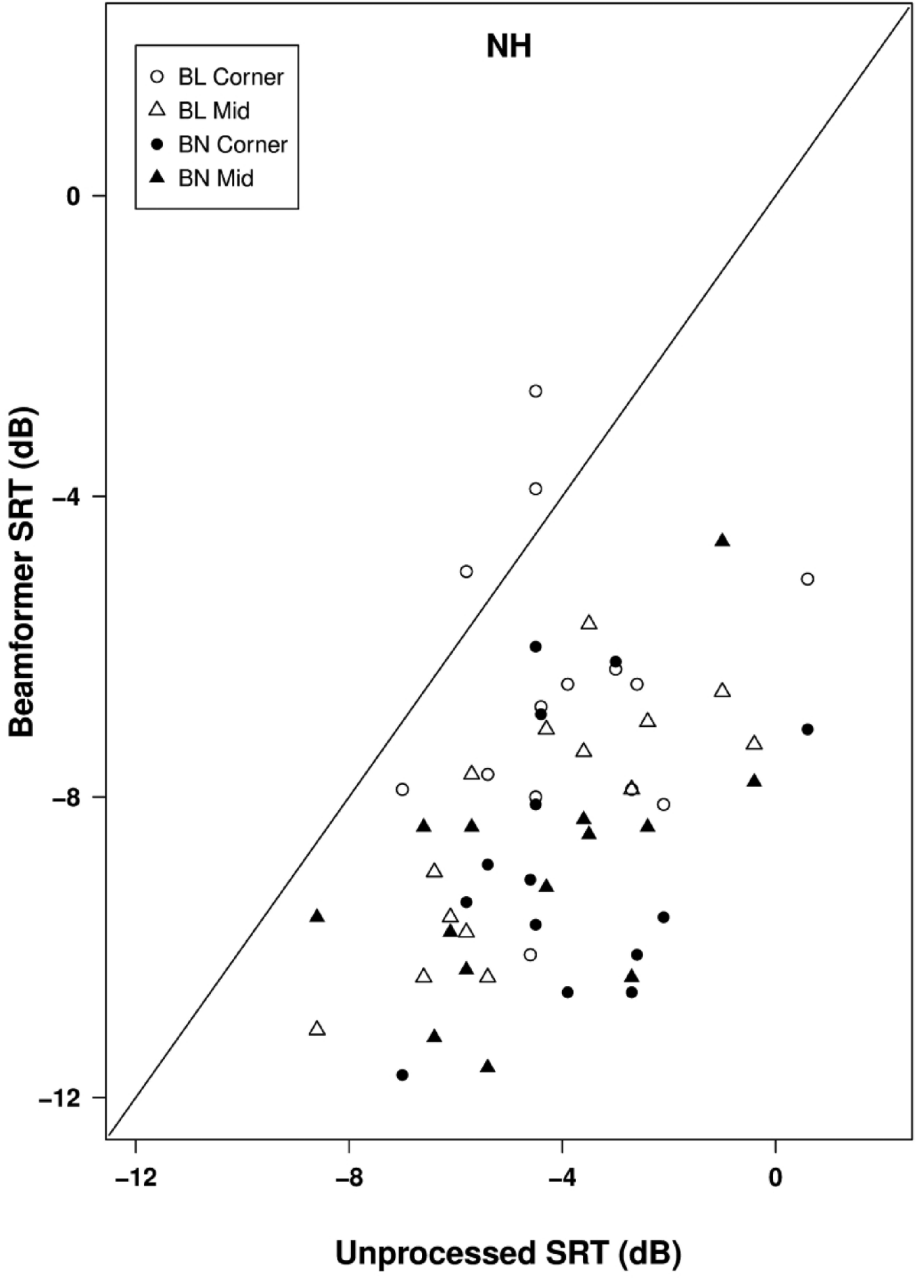
Scatterplot of SRTs in the classroom environment for normally-hearing listeners. SRTs for beamformer alone conditions are plotted against those for unprocessed conditions. Points below the line of equality indicate a beneficial effect of beamforming.

*Normal Hearing listeners.* Figure 8 shows the SRTs for each processing type in the simulated classroom environment for locations C (left panel) and M (right panel). As for HI listeners, performance was slightly poorer in the corner location, but the pattern across processing conditions was similar for both locations. The mean SRTs for unprocessed stimuli were -3.9 dB for location C and -4.5 dB for location M. These values are respectively 7.8 dB and 6.8 dB lower than the values observed with HI listeners. For location C, the benefit to mean SRT of applying a beamformer alone was 5.0 dB for the binaural case and 2.8 dB for the bilateral case. For location M, the benefits were 4.6 dB (binaural) and 3.9 dB (bilateral). Individual data are shown in Figure 9.

**Figure 10.**
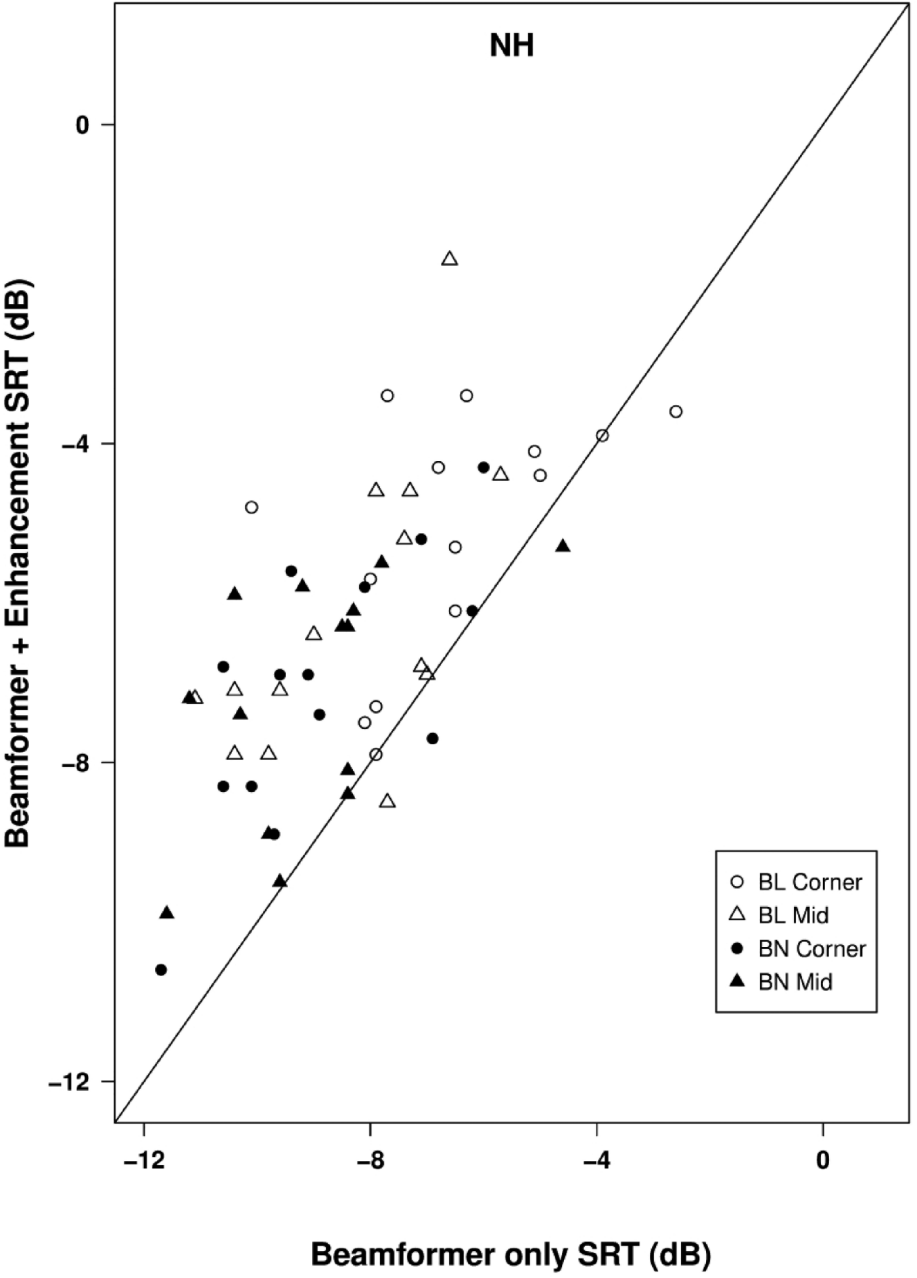
Scatterplot of SRTs in the classroom environment for normally-hearing listeners. SRTs for beamformer plus mask-informed enhancement are plotted against those for beamformer alone. Points below the line of equality indicate a beneficial effect of speech enhancement.

For both types of beamformer and both locations, adding the mask-informed enhancement worsened mean SRTs compared to beamformer alone by between 1.5 dB (BL corner) and 2.2 dB (BL middle), slightly greater than the deficits observed with HI listeners. Effects of adding mask-informed enhancement for individual listeners are shown in Figure 10. In the other enhancement conditions performed with NH listeners there were only very small differences in mean SRT relative to the binaural beamformer alone. For Kalman filtering mean SRTs were better than beamforming alone by 0.1 dB in location C and 0.2 dB in location M. For Wiener filtering there was a deficit of 0.6 dB in location C and a benefit of 0.2 dB in location M.

An ANOVA with location as a between-subjects factor and all seven processing conditions treated as levels of a single within-subjects factor showed a significant main effect of processing type [*F*(6,156) = 70.74, *p* < 0.001], no significant effect of location [*F*(1,26) = 2.01, *p* = 0.168], and no significant interaction [*F*(6,156) = 1.80, *p* = 0.102]. Bonferroni-corrected pairwise comparisons showed that: performance in all processed conditions was better than that in the unprocessed case; the binaural beamformer outperformed the bilateral beamformer, both with and without the mask-informed enhancement; addition of mask-informed enhancement was detrimental for both types of beamformer; and performance with the binaural beamformer and mask-informed enhancement was poorer than that in the three other conditions with a binaural beamformer, which did not differ from each other.

As with the HI data, an additional ANOVA was performed including only BL, BN, BL+M and BN+M processing conditions, together with location as a between-subjects factor (Table 4). Consistent with the HI data this analysis showed highly significant effects of beamformer type and the presence of speech enhancement, although some differences were observed. For NH listeners the main effect of location was not significant and there was no significant interaction between beamformer type and the presence of speech enhancement. There was, however, a significant interaction between beamformer type and location. Supplementary 2-way ANOVAs showed that beamformer type was significant for each location analysed separately, although the advantage for binaural over bilateral was smaller in the middle location.

**Table 4.** Mixed ANOVA on SRTs of normal hearing listeners in conditions with beamforming and mask-informed enhancement in the simulated classroom. Significant p values at the 5% level are shown in bold.

Factor	df	F	p
3-way ANOVA			
Beamformer Type	(1,26)	73.50	**<0.001**
Enhancement	(1,26)	115.24	**<0.001**
Location	(1,26)	1.98	0.172
Beamformer Type x Enhancement	(1,26)	0.00	0.982
Beamformer Type x Location	(1,26)	13.15	**0.001**
Enhancement x Location	(1,26)	1.26	0.273
Beamformer Type x Enhancement x Location	(1,26)	0.49	0.490
Corner Location 2-way ANOVA			
Beamformer Type	(1,13)	67.26	**<0.001**
Enhancement	(1,13)	48.71	**<0.001**
Beamformer Type x Enhancement	(1,13)	0.23	0.641
Middle Location 2-way ANOVA			
Beamformer Type	(1,13)	13.69	**0.003**
Enhancement	(1,13)	66.86	**<0.001**
Beamformer Type x Enhancement	(1,13)	0.26	0.617

## Discussion

The main focus of the study was to assess the effects of a combination of beamforming and mask-informed speech enhancement on the speech recognition performance of HI listeners in a simulated realistic adverse listening environment. The two main findings were that 1) relative to the unprocessed condition, benefits to speech recognition were significantly greater for binaural beamforming than bilateral beamforming, and 2) applying mask-informed speech enhancement in combination with binaural beamforming resulted in poorer performance than applying the beamformer alone. As might be expected, performance overall was substantially better for NH than for HI listeners, but the pattern of effects across processing conditions was similar across the two types of listener. Similarly, while performance overall was better for the middle than the corner location, differences between processing conditions were broadly similar for the two positions in the simulated classroom.

The finding that binaural beamforming produced lower SRTs than bilateral beamforming for HI listeners, on average by around 1–2 dB, shows that in the situation tested here, in which target speech was presented from a fixed frontal location against a background of classroom noise, the improvement in SNR provided by a narrower beam outweighed the loss of spatial diversity in the residual noise. This is largely consistent with previous comparisons of binaural and bilateral beamforming (e.g., Cornelis, Moonen, & Wouters, 2012; Moore et al., 2019; Neher, Wagener, & Latzel, 2017; Völker, Warzybok, & Ernst, 2015). The size of the effect of type of beamformer was similar for NH listeners which suggests that the advantage for binaural beamforming did not arise due to limitations on the spatial hearing abilities of HI listeners rendering them unable to take advantage of any useful spatial cues that may have been preserved in the bilateral case. However, it should be noted that the beamformers here were optimally adapted for the target source and that it remains possible that for HA users in real-world settings there may be situations in which preserving the spatial diversity of the residual noise is beneficial. The appropriate balance between improving SNR and preserving spatial cues may also depend upon the nature of the competing sounds.

Neher et al. (2017) measured recognition of sentences from a target talker at 0° azimuth in different masking conditions for several directional processing algorithms which varied the trade-off between SNR improvement and preservation of spatial information. In a background of spatially diffuse cafeteria noise, performance was best with processing that provided maximal SNR improvement. However, in conditions with two symmetrically located competing talkers, processing which preserved low-frequency spatial cues performed better. Wang et al. (2020) also found evidence of a benefit of preserving spatial information for sentence recognition with a frontal target talker presented against a small number of symmetrically distributed competing talkers. The current results, then, contribute to a pattern of findings which suggests that maximising the improvement in SNR, even at the expense of spatial cues in the residual noise, may be an optimal approach for spatially diffuse noise backgrounds, while preservation of spatial cues may be of benefit in situations with a small number of competing sound sources, at least for those HA users with sufficient spatial hearing ability (Neher et al., 2017). This influence of noise condition emphasises the need for evaluations of HA processing algorithms to employ test scenarios that are realistic and representative of situations typically faced by HA users in the real world. Further aspects of real world communication not assessed here that may influence the optimal balance between SNR improvement and preservation of spatial information include listener head movements, dynamic changes in the identity and location of the talker of interest and the availability of visual information.

In most of the conditions tested here, the addition of mask-informed enhancement to beamforming resulted in a small deficit in performance compared to that with beamforming alone, although for HI listeners the deficit was not significant for the bilateral beamformer. Two alternative forms of enhancement, Wiener filtering and Kalman filtering, which were tested only in conjunction with binaural beamforming and only in NH listeners, performed significantly better than mask-informed enhancement but had no effect on performance relative to that with beamformer alone. One limitation on the effectiveness of the mask-informed enhancement implemented here may be that the noise used in training of the DNN was not sufficiently similar to that in which testing took place. It is possible that training with a wide range of noise types might allow mask-informed enhancement to improve performance beyond that achieved with beamforming alone. Two other factors may contribute to the small deficit seen with mask-informed enhancement. First, it is well-known that modifications applied by speech enhancement algorithms to the speech spectral magnitude in the TF domain generally degrade SRT for NH listeners, even though opinion-based quality improvements are obtained (e.g., Hilkhuysen, Gaubitch, Brookes & Huckvale, 2012; Hu & Loizou, 2007). The current results imply that such modifications also degrade intelligibility for HI listeners. Second, the interaural level-difference and interaural time-difference spatial cues in the mask-informed processing are preserved by ensuring that the left and right channels are modified with the same gain. However, this is ensured independently at each TF region without considering the spectrotemporal context. Further testing is required to investigate whether this approach degrades the spatial information and the consequent SRM and, accordingly, gives rise to the loss of SRM seen in the results. Further research might also examine performance in conditions in which the beamformer is not optimised for the true target position, since this optimisation may limit the potential for mask-informed enhancement to provide additional benefit. Finally, it would also be of interest to compare the performance of the processing methods used here to that of other approaches to integrating spatial filtering and speech enhancement (e.g., Andersen et al, 2021; Koutrouvelis, Hendriks, Heusdens & Jensen, 2017).

## Conclusions

For both NH and HI listeners tested in a simulated adverse listening environment that provided realistic auditory spatial information, greater benefit to sentence recognition was provided by binaural beamforming than by bilateral beamforming. This suggests that, at least for optimally adapted beamformers, the greater improvement in SNR provided by using all available microphones to derive a narrower beam outweighed any benefit from the preservation of spatial cues in the residual noise. Combining these optimally adapted beamformers with a type of DNN-based mask-informed speech enhancement did not improve performance compared to beamforming alone, and in most cases made performance slightly poorer. Additional testing performed solely in NH listeners showed that for speech enhancement combined with binaural beamforming, both Wiener filtering and Kalman filtering produced better performance than the mask-informed technique implemented here, but neither method improved performance relative to beamforming alone.
